# ^188^Re-SSS/Lipiodol: Development of a Potential Treatment for HCC from Bench to Bedside

**DOI:** 10.1155/2012/278306

**Published:** 2012-02-22

**Authors:** Nicolas Lepareur, Valérie Ardisson, Nicolas Noiret, Etienne Garin

**Affiliations:** ^1^Centre Régional de Lutte Contre le Cancer Eugène Marquis, INSERM UMR-S 991, Avenue de la Bataille Flandres-Dunkerque, CS 44229, 35042 Rennes, France; ^2^Ecole Nationale Supérieure de Chimie de Rennes, CNRS UMR 6226, Avenue du Général Leclerc, CS 50837, 35708 Rennes, France; ^3^Université Européenne de Bretagne, 35000 Rennes, France

## Abstract

Hepatocellular carcinoma (HCC) is the 5th most common tumour worldwide and has a dark prognosis. For nonoperable cases, metabolic radiotherapy with Lipiodol labelled with *β*-emitters is a promising therapeutic option. The Comprehensive Cancer Centre Eugène Marquis and the National Graduate School of Chemistry of Rennes (ENSCR) have jointly developed a stable and efficient labelling of Lipiodol with rhenium-188 (E_*β*max_ = 2.1 MeV) for the treatment of HCC. The major “milestones” of this development, from the first syntheses to the recent first injection in man, are described.

## 1. Introduction

Hepatocellular carcinoma (HCC) is the fifth most common tumour worldwide and even ranks second in terms of mortality [1, 2]. Moreover, only a small number of cases are eligible to curative treatments, such as resection or transplantation. For the rest, a wide range of palliative treatments can be proposed, among which are chemoembolisation and radioembolisation with Lipiodol [3–6]. Lipiodol is an oily medium which has shown to be selectively retained in tumour when administered intra-arterially [7]. Lipiodol has been labelled with iodine-131 [8, 9], rhenium-188 [10, 11], yttrium-90 [12, 13], holmium-166 [14], and lutetium-177 [15]. Some early trials with phosphorus-32 have also been described [16]. However, to date, only the first two have been used in man, the iodine-131-labelled Lipiodol (Lipiocis) having a market authorisation. ^188^Relabelled Lipiodol seems the most promising one, being able to circumvent the major drawbacks of iodine-131 (long half-life, medium beta energy, strong gamma energy, and cost). Indeed, rhenium-188 has ideal properties for molecular radiotherapy (E*β*_max_ = 2.1 MeV with a maximum tissue penetration of 11 mm, E*γ* = 155 keV (15%), *t*_1/2_ = 17 h) and has the added advantage of being available on a cost-effective day-to-day basis thanks to its generator mode of production [17]. It has thus attracted much interest [18, 19].

First attempts to label Lipiodol with radioisotopes other than iodine-131 were done with a covalently bond chelate, with disappointing results [12, 20]. It was thus postulated that solubilisation of a lipophilic chelate into Lipiodol would make a suitable “radiolabelling” [21]. Soon after, several teams investigated this promising approach [22, 23]. It is in that context that the Centre Eugène Marquis and the Ecole Nationale Supérieure de Chimie de Rennes (ENSCR) decided to combine respectively their experience on radioembolisation of HCC with ^131^I-Lipiodol [9, 24–27] and knowledge of technetium and rhenium coordination chemistry [28–31], to label Lipiodol with a ^188^Rechelate of their own design. 

## 2. Synthesis

### 2.1. ^185/187^Re/^99^Tc Syntheses

**Figure 1:**
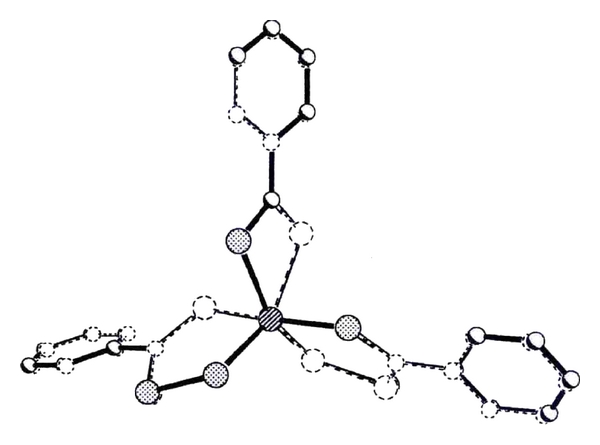
Superposition of the crystal structures of ^185/187^Re-SSS and ^99^Tc-SSS complexes (reprinted from: N. Lepareur, Ph.D. thesis no. 2003 REN 10110, 2003).

Previous studies at the ENSCR led to the preparation of a new class of complexes with perthiobenzoate and dithiobenzoate moieties [M (PhCS_3_)_2_ (PhCS_2_)] which structure is given in Figure 1, both with rhenium [32] and with technetium-99 [33]. These complexes were nicknamed SSS, standing for “Super-Six sulphur”, because the metal core is coordinated by six sulphur atoms. One of the interests of these complexes is that the metal is at the oxidation state +III, which is more stable than +V and they are, in addition, susceptible to bifunctional approach to design target-specific agents [31, 34].

### 2.2. ^99m^Tc Synthesis

**Figure 2:**
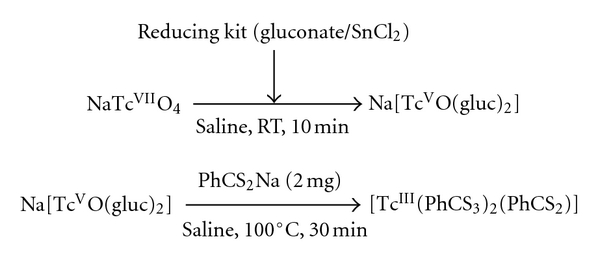
Synthesis of ^99m^Tc-SSS.

The SSS complex was subsequently prepared with technetium-99 m, using a freeze-dried kit method (containing 0.75 mg tin chloride, 75 mg calcium gluconate, and 25 mg sodium chloride, diluted 1/10), initially in a view of lymphocytes labelling [35]. The complex was obtained, according to procedure shown in Figure 2, with good yield and good radiochemical purity (>95%). Moreover, it proved to be very stable and to be quite lipophilic (log *P* = 3.33).

It was, therefore, a good candidate to label Lipiodol. The method previously described by Jeong et al. [22] was used, that is, once the complex is synthesised, 2-3 mL of Lipiodol Ultra-Fluide (simply called Lipiodol) is added. The mixture is shaken then centrifuged at 2200 g for 10 min, and the phases are carefully collected. ^99m^Tc-SSS/Lipiodol is thus obtained with a 96 ± 2.8% yield and a final RCP of 92.5 ± 2.6% [36]. The preparation showed satisfactory reproducibility, and the labelling proved to be stable.

### 2.3. ^188^Re Synthesis

**Figure 3:**
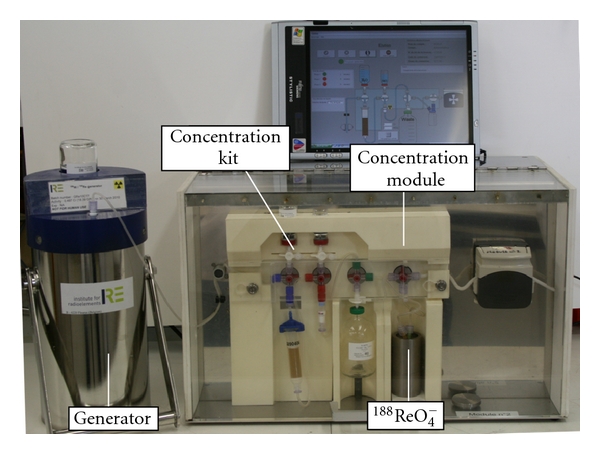
^188^W/^188^Re generator and its remote-controlled elution/concentration system (IRE, Fleurus, Belgium).

Rhenium-188 is obtained in the form of perrhenate by elution of a ^188^W/^188^Re generator, similar to the ^99^Mo/^99m^Tc generator. The major difference lies in the necessity of postelution concentration to obtain high-volumic activity (up to 20 GBq/mL with a 37 GBq generator), due to the lower specific activity of ^188 ^W compared to ^99^Mo, necessitating a bigger alumina column (see Figure 3). First trials to prepare ^188^Re-SSS proved to be disappointing. Indeed, rhenium-188, though its chemistry is very close to that of technetium-99 m, is much harder to reduce and tends to reoxidise very quickly. It requires harsher reaction conditions. As a consequence, reaction conditions as well as kit composition had to be modified [37]. Amounts and type of reducing agent were varied as well as ancillary ligand. Antioxidants and chelating agents were added. Volume, pH, heating temperature, and reaction time were also modulated. Eventually, the solution came with the adjunction of potassium oxalate, which eases the reduction of perrhenate by expanding its coordination sphere, as shown by Boschi et al. [38]. Finally, the optimal procedure was determined as

Kit formulation: 0.8 mg SnCl_2_·2H_2_O (dissolved in 0.1 mL HCl 1 M), 7.5 mg sodium gluconate, 30 mg ascorbic acid, and 40 mg potassium oxalate. This freeze-dried kit is reconstituted in 0.5 mL saline, and the perrhenate (0.5 mL of saline) is then added. After 15 min at room temperature, 20 mg of sodium dithiobenzoate is added, and the solution is heated for 30 min at 100°C, to provide the ^188^Re-SSS complex, as a precipitate. 2-3 mL of Lipiodol is added to the mixture, which is then centrifuged, as previously described with technetium-99 m.^ 188^Re-SSS/Lipiodol was obtained with a 87 ± 9.1% yield and a final RCP of 93 ± 3.4%. Labelling was further optimised to reach 97.3 ± 2.1% yield and a final RCP of 94.1 ± 1.7% [39].

### 2.4. High-Activity Upgrade and Automation

A new efficient and stable labelling of Lipiodol was described. However, for activities above 1850 MBq (therapeutic activities), the labelling yield decreased dramatically. Consequently, the synthesis had to be further improved, in view of HCC treatment. Kit formulation was tuned, and reaction conditions were slightly modified.

A freeze-dried kit (4 mg SnCl_2_·2H_2_O (dissolved in 0.1 mL HCl 1 M), 30 mg sodium gluconate, 30 mg ascorbic acid, 40 mg potassium oxalate) is reconstituted in 0.5 mL saline, and the perrhenate (0.5 mL of saline) is then added. After 15 min at room temperature, 40 mg of sodium dithiobenzoate is added, and the solution is heated for 15 min at 100°C, to provide the ^188^Re-SSS complex, as a precipitate. 2-3 mL of Lipiodol is added to the mixture, which is then stirred with a vortex. After 10 min of centrifugation (2200 g), both phases are separated, and the lower phase (radiolabelled Lipiodol) is carefully recovered. ^188^Re-SSS/Lipiodol is obtained with a 98.56 ± 1.2% yield and a final RCP of 92.52 ± 2.3% and is stable for at least 7 days [40].

**Figure 4:**
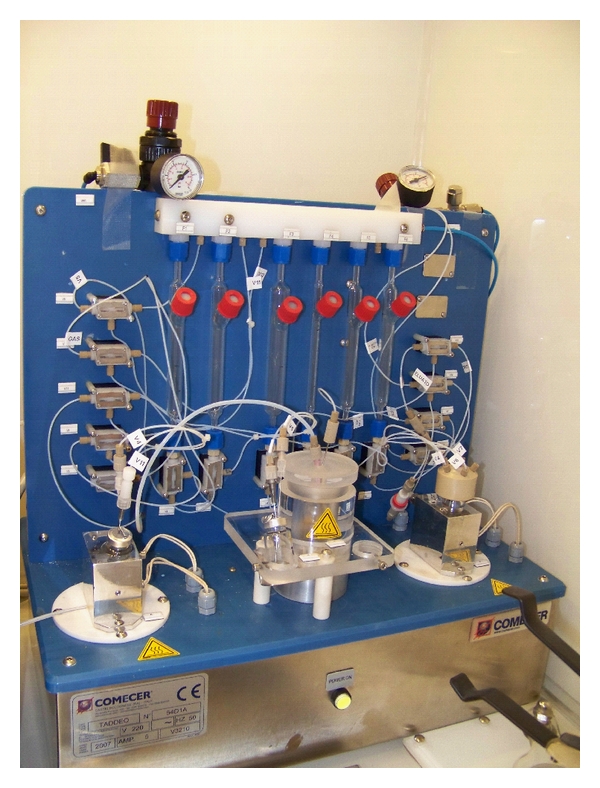
TADDEO module (COMECER, Castel Bolognese, Italy).

**Figure 5:**
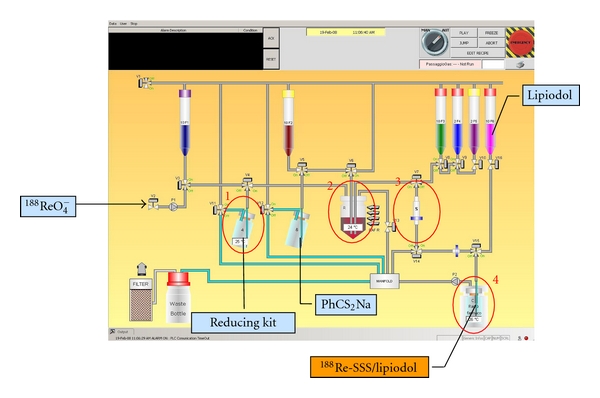
Flowchart of the TADDEO module for the preparation of ^188^Re-SSS/Lipiodol.

Having to handle high activities of ^188^Re to prepare therapeutic doses can result in an excessive radiation exposure to the operator, and particularly at the finger tips [41]. The authors have thus developed an automated procedure to limit the radiation exposure to the personnel, as well as to have a reproducible synthesis, in view of clinical trials [40]. The remote-controlled system employed is a TADDEO module (COMECER, Castel Bolognese, Italy) and is displayed in Figure 4. The radiolabelling procedure was once more adapted to be automated. The main change was the substitution of the centrifugation step and the use of solid-phase extraction cartridges to purify the product. The final yield is somewhat lower than with manual preparation (52.68 ± 9.6%), due to the loss of activity in the tubing and the vessels (Figure 5).

**Figure 6:**
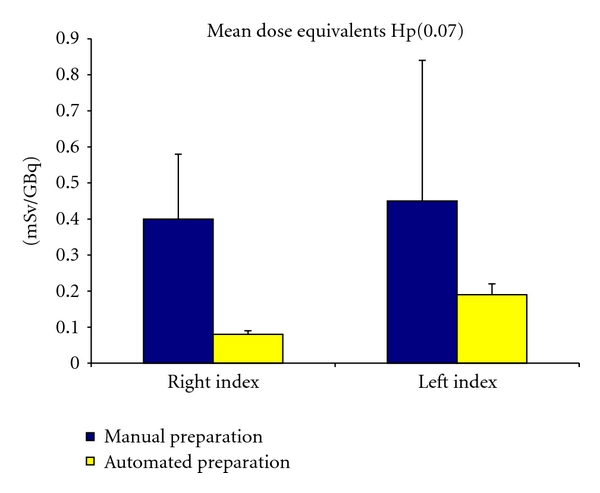
Mean dose equivalents (mSv/GBq) for both hands for manual (*n* = 3) and automated (*n* = 2) preparations of ^188^Re-SSS/Lipiodol, measured with TLD fixed at the tips of the indexes.

The foremost gain of the automation was in the dose received by the operator. This is particularly true for the dose to the extremities. Impact of the automation was studied with thermoluminescent dosimeters (TLD) fixed at the finger tips, and, respectively, 80 and 58% decreases in the right-hand and left-hand doses were shown [42]. Personal dose equivalents—measured with continuously readable EPD dosimeters—were reduced by 38 and 43% for Hp (10) and Hp (0.07), respectively. Mean dose equivalents (mSv/GBq) for both hands are displayed in Figure 6.

## 3. Preclinical Studies

Good targeting and stability of radiolabelled Lipiodol were investigated *in vivo*, in healthy pigs, then in hepatoma-bearing rats. The radiotracer was injected through the hepatic artery, and biodistribution was checked by scintigraphy and *ex vivo* countings. Autoradiography was also done, to assess more precisely the fixation of the radiotracer.

### 3.1. Healthy Pigs

**Figure 7:**
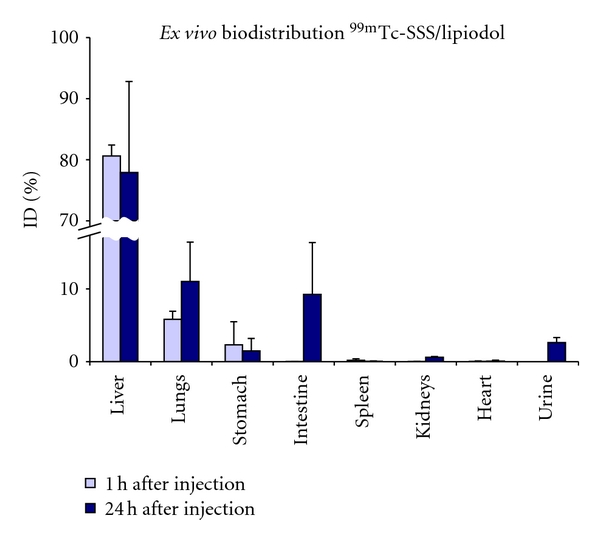
*Ex vivo* biodistribution of ^99m^Tc-SSS/Lipiodol in healthy pigs (*n* = 2 for each time point).

^99m^Tc-SSS/Lipiodol could be useful for carrying pretherapeutic dosimetry studies, as is performed with ^99m^Tc-MAA for ^90^Y-labelled microspheres [43]. It was injected into the hepatic artery of healthy pigs and showed a biodistribution pattern similar to that of ^131^I-labelled Lipiodol in human [36]. It has a preferential liver uptake, as shown in Figure 7. Fixation was stable and showed only mild digestive elimination 24 h after injection.

**Figure 8:**
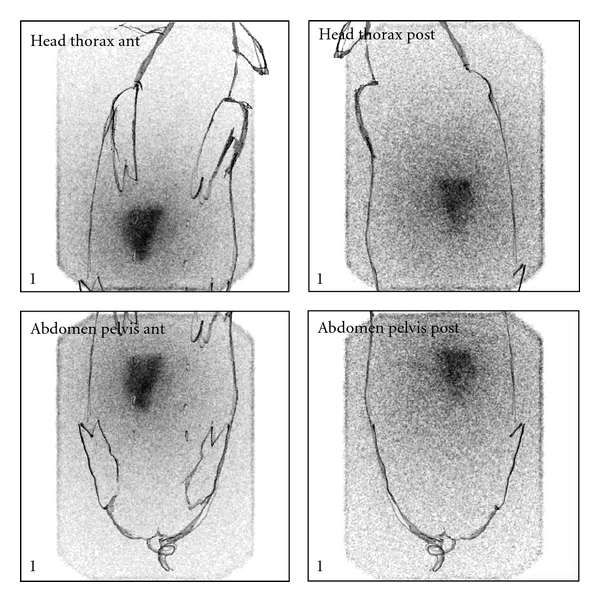
Scintigraphic scans of ^188^Re-SSS/Lipiodol 48 h after intra-arterial injection in healthy pigs.

**Figure 9:**
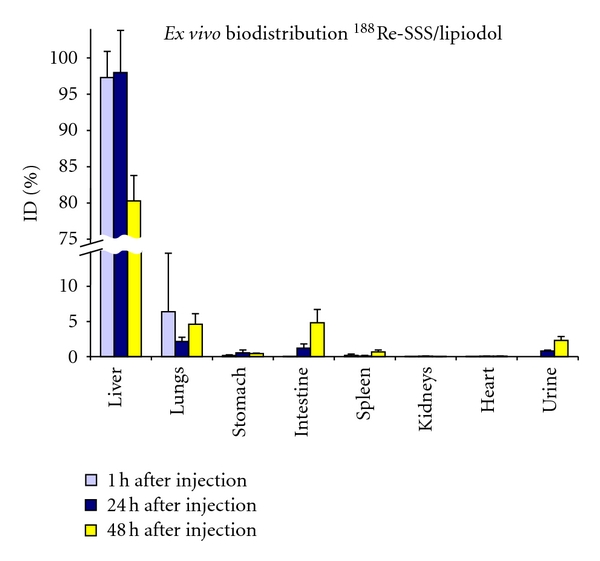
*Ex vivo* biodistribution of ^188^Re-SSS/Lipiodol in healthy pigs (*n* = 2 for each time point).

**Figure 10:**
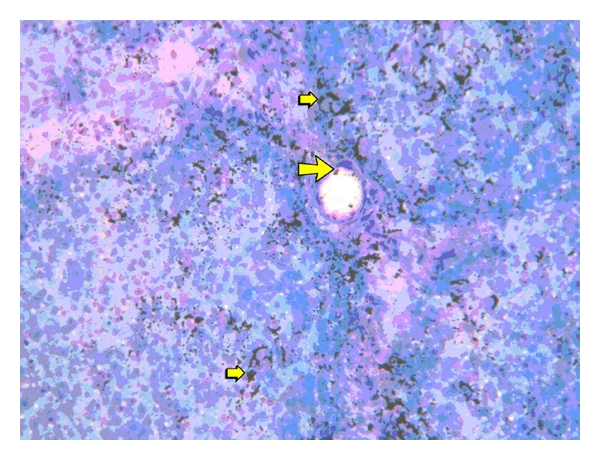
Fused autoradiography/histology tissue stain of the liver, 1 h after intra-arterial injection of 30 MBq of ^188^Re-SSS/Lipiodol. Radioactivity (black spots) is weakly noticeable in the region of hepatic artery and portal space (big yellow arrow) and strongly in capillary sinusoids (small yellow arrow), May Grumwald Giemsa coloration,  ×40.

^188^Re-SSS/Lipiodol was also investigated in healthy pigs [44]. Scintigraphic scans (Figure 8) and *ex vivo* countings (Figure 9) show the quasiexclusive hepatic fixation, with a slight pulmonary uptake (not visible on scintigraphy). The fixation also proved to be stable with a very weak urinary and intestinal elimination. At the microscopic level, the radioactivity is mainly—and rapidly—located in the sinusoids (Figure 10), as Lipiodol alone [45], where it is retained.

### 3.2. HCC-Bearing Rats

Unfortunately, no porcine model of hepatocarcinoma was described, and attempts to develop one with human hepatocarcinoma cells in immunodepressed pigs with cyclosporine gave no results. On the contrary, murine hepatoma models are well documented. It was thus decided to investigate the tumour uptake of ^188^Re-SSS/Lipiodol in rats inoculated with N1S1 hepatocarcinoma cell line [39]. Our team, in collaboration with J. P. Benoit's team in Angers, developed a new technique for the tumoral inoculation as well as the intra-arterial injection [46]. 

**Figure 11:**
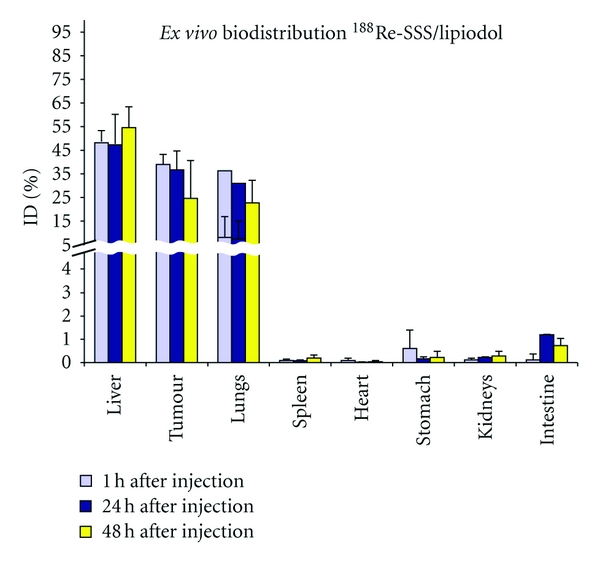
*Ex vivo* biodistribution of ^188^Re-SSS/Lipiodol in hepatoma-bearing rats (*n* = 3 for each time point).

Results showed preferential hepatic uptake, with a weak to moderate pulmonary uptake, and, most importantly a good tumour retention (Figure 11). This is consistent with the other ^188^Relabelled Lipiodol methods. The tumour-to-liver ratio increases from 2.9 ± 1.5 to 4.1 ± 0.7 between 1 h and 48 h. However, this model has its limitations, notably the fact that a single small tumour in a rat is probably too small for rhenium-188 to be truly effective. Indeed, when compared to ^131^I-lipiodol, the latter proved to be more effective [47].

### 3.3. Toxicology Studies

To assess the safety of the radiotracer, a toxicity study—acute and chronic—has been undertaken in dogs (Beagles), with the nonradioactive analogue ^185/187^Re-SSS/Lipiodol, prepared in the same conditions as for clinical preparation (sterile GMP kits, same amounts of reactants, remote-controlled procedure). The Re-SSS/Lipiodol was injected for less than 24 h after preparation.

**Figure 12:**
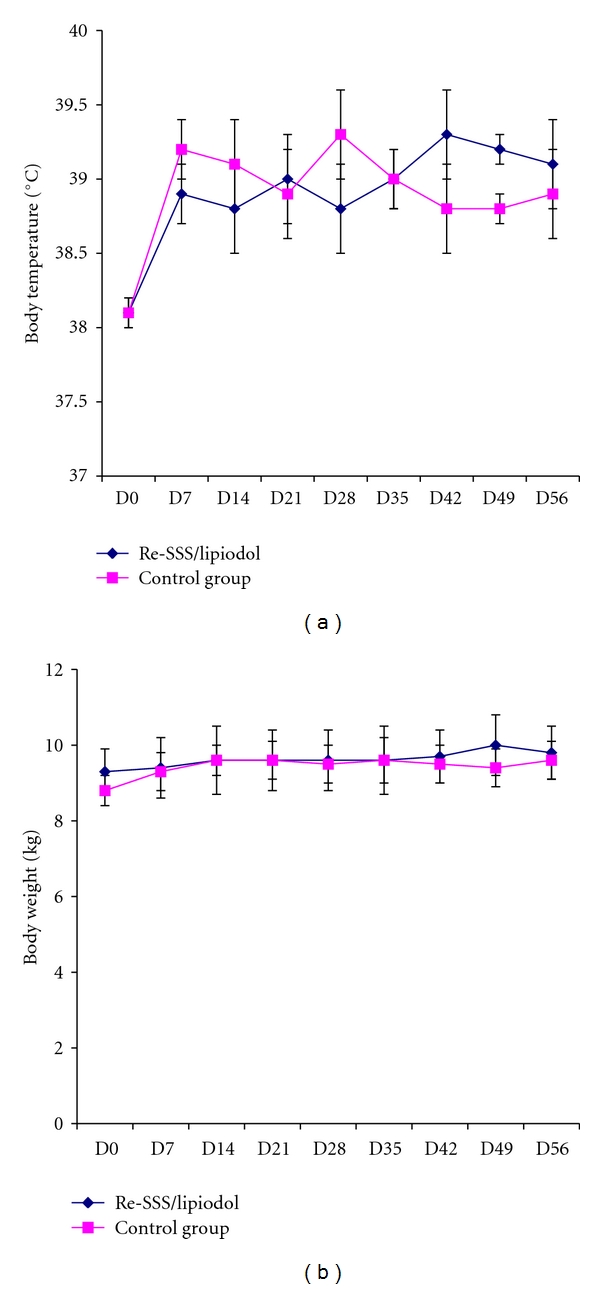
Physiological parameters; toxicity study in dogs (*n* = 4 for each group).

**Figure 13:**
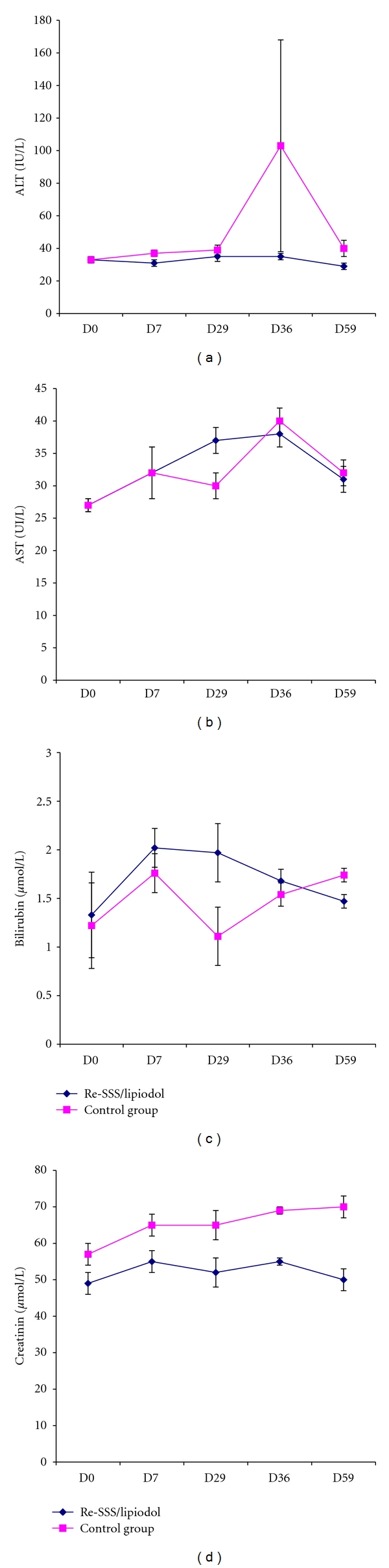
Some clinical chemistry values; toxicity study in dogs (*n* = 4 for each group); ALT (alanine transaminase), AST (aspartate transaminase), and bilirubin are biomarkers of liver function; creatinin is a marker of renal function.

The study comprised two phases. For the first phase (7 days), animals (3 males + 3 females) received a single injection at D1, and for the second one, animals (2 males + 2 females) received one dose at D1 and one at D30. Control group (5 males + 5 females) received Lipiodol alone. Some results are summarised in Figures 12 and 13. No change related to the parenchyma of organs or at the site of injection has been detected, either at D7 or at D59.

This study thus demonstrated lack of toxicity of Re-SSS/Lipiodol, opening the way for the injection in human.

## 4. Clinical Investigation

**Figure 14:**
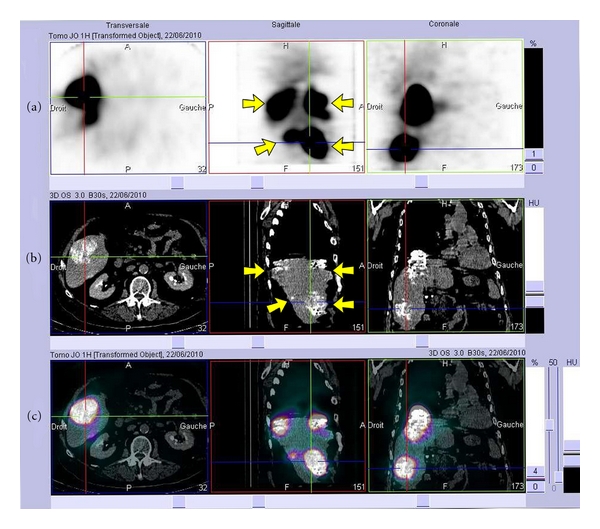
Scintigraphy (a) showing intense hyperfixation (in black) in 4 tumour foci, CT scan (b) showing intense Lipiodol retention (in white) in the nodules and fusion image (c) of scintigraphy and CT scan; 71-year-old male, 1 h after injection of 1.85 GBq of ^188^Re-SSS/Lipiodol. Transverse, sagittal, and coronal views.

Files were submitted to the relevant authorities, that is, French Agency for the Safety of Health Products (AFSSAPS), French Nuclear Safety Authority (ASN), and Ethical Committee (*Comité de Protection des Personnes*, CPP), and approval was eventually granted. A phase 1 escalation dose study was thus initiated (Lip-Re-1, EudraCT no. 2009-013231-37). This study will comprise 4 dose stages, ranging from 1.85 to 7.4 GBq. Each stage will comprise 3 to 6 patients, depending on the toxicity (or lack thereof) of the compound. To date, 5 patients have been injected (3 with 1.85 GBq and 2 with 3.7 GBq). For illustration, SPECT/CT scans of the third patient 1 h after injection of 1.85 GBq of ^188^Re-SSS/Lipiodol are shown in Figure 14, displaying the good targeting of the radiopharmaceutical candidate (tumour-to-nontumour max ratio = 15). 4 nodules are clearly visible on the sagittal plane. This patient (71-year-old male, with multifocal HCC, in progression after Sorafenib treatment) responded well to the treatment, and his disease was stabilised for a couple of months.

## 5. Conclusion

In conclusion, we have developed a potential HCC treatment by radioembolisation, with a phase 1 clinical trial currently in progress. This represents almost ten years of multidisciplinary research, from basic chemistry to clinic. Its clinical relevance has now to be demonstrated, and its efficiency and tolerance have to be compared to other existing therapeutic options.
